# Supplementary material for: Venomix: a simple bioinformatic pipeline for identifying and characterizing toxin gene candidates from transcriptomic data
Source: PeerJ. 2018 Jul 31;6:e5361. doi: 10.7717/peerj.5361 (PMC6074769; doi:10.7717/peerj.5361)
Supplement: Supplemental Information 6 [file peerj-06-5361-s006.gz › FinalOutput_1E-6/Glutaminyl-peptide_cyclotransferase_1/finaltree.pdf]

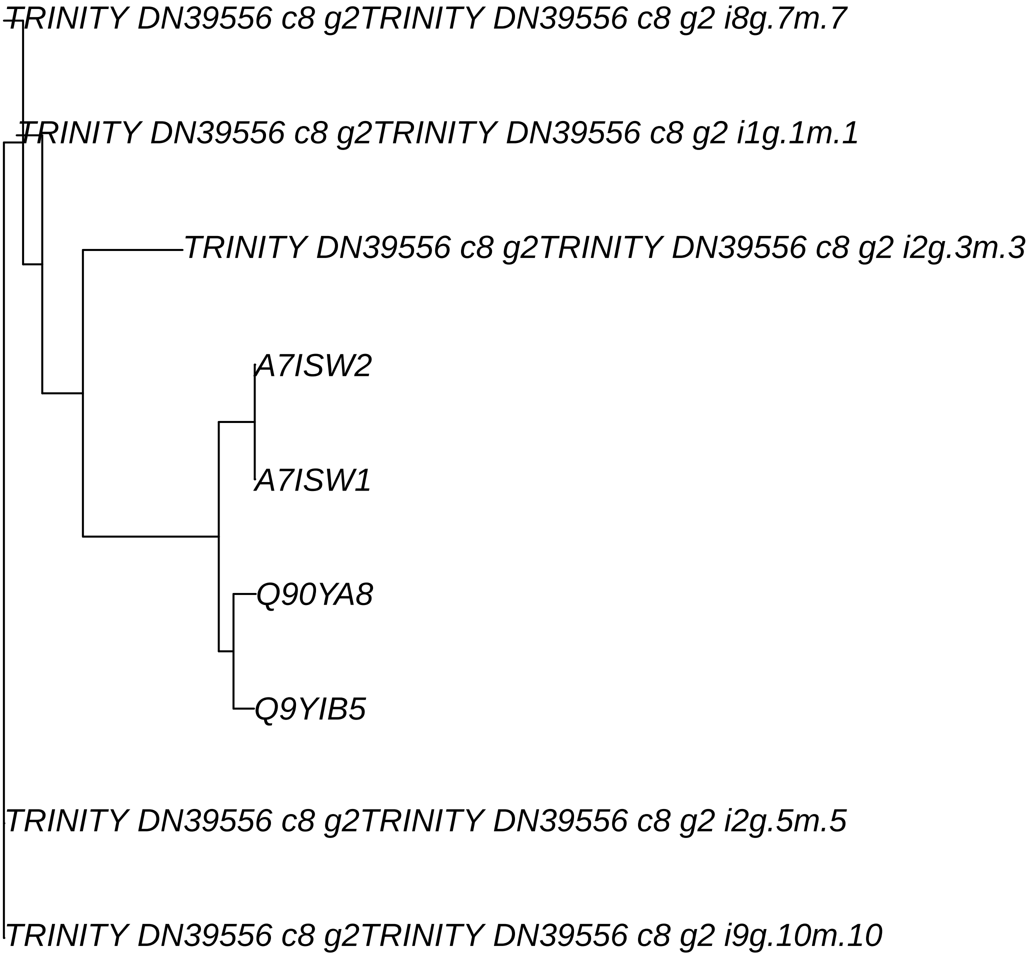
TRINITY DN39556 c8 g2TRINITY DN39556 c8 g2 i8g.7m.7
TRINITY DN39556 c8 g2TRINITY DN39556 c8 g2 i1g.1m.1
TRINITY DN39556 c8 g2TRINITY DN39556 c8 g2 i2g.3m.3
A7ISW2
A7ISW1
Q90YA8
Q9YIB5
TRINITY DN39556 c8 g2TRINITY DN39556 c8 g2 i2g.5m.5
TRINITY DN39556 c8 g2TRINITY DN39556 c8 g2 i9g.10m.10
